# Modeling and Experimental Validation of Forces in Low-Frequency Vibration-Assisted Drilling Considering Bone Anisotropic Effects

**DOI:** 10.3390/ma19194186

**Published:** 2026-09-30

**Authors:** Ying Han, Jun Wang, Xianzheng Zhou, Yimiao Chen, Qinhe Zhang

**Affiliations:** 1 Department of Cloud Computing Technology and Applications, Shandong University of Commerce and Technology, Jinan 250103, China; 2 Key Laboratory of Efficient and Clean Machinery Manufacturing of Ministry of Education, School of Mechanical Engineering, Shandong University, Jinan 250100, China; 3 National Demonstration Center for Experimental Mechanical Engineering Education, Shandong University, Jinan 250100, China

**Keywords:** low-frequency vibration-assisted drilling, bone drilling, drilling force modeling, bone anisotropy, experimental validation

## Abstract

**Highlights:**

- A mechanistic force model for low-frequency vibration-assisted bone drilling is established, considering main cutting lips, chisel edge and asymmetric chip thickness.
- A direction-dependent coefficient is embedded to quantitatively characterize cortical bone anisotropy instead of having a qualitative description only.
- LVAD reduces drilling force by up to 16.52% compared to conventional drilling; yz-plane drilling produces larger forces than that on the xz-plane.
- The model achieves high prediction accuracy with *R*^2^ = 0.94–0.97 and RMSE < 3.2 N under limited drilling depth without chip clogging.

**Abstract:**

This study focuses on modeling and experimental validation of drilling forces in low-frequency vibration-assisted bone drilling (LVAD), with explicit consideration of bone anisotropic effects. A mechanistic force model is established by integrating contributions from the main cutting edges and chisel edge, and a direction-dependent anisotropic coefficient is introduced to characterize the anisotropic shear strength of cortical bone. The influences of feed rate, spindle speed, drill diameter, vibration amplitude, frequency, and drilling orientation on drilling forces are analyzed theoretically and experimentally. Results show that drilling forces increase with feed rate and drill diameter and decrease with spindle speed and vibration amplitude. LVAD reduces drilling forces by up to 16.52% compared with conventional drilling. Perpendicular drilling produces lower forces than oblique drilling, and forces in the yz-plane are higher than those in the xz-plane due to bone anisotropy. The proposed model is well verified by experiments, providing a theoretical basis for parameter optimization in clinical bone drilling.

## 1. Introduction

Bone drilling is widely applied in numerous surgical procedures, including orthopedics, neurosurgery, and dentistry [1]. However, due to the anisotropic structure and biological properties of cortical bone, excessive drilling forces generated during the drilling process can induce microcracks on the drilled surface, causing mechanical damage to the bone tissue. Moreover, the fixation process of implants can lead to the propagation of these microcracks, resulting in implant loosening [2,3], which compromises surgical outcomes and hinders postoperative recovery for patients. Drilling forces also affect process stability, surface quality, drilling temperature, and tool wear, making them a critical concern in surgical bone machining.

During vibration-assisted machining, periodic separation between the cutting tool and the workpiece enables intermittent cutting. Compared with conventional drilling (CD), low-frequency vibration-assisted drilling (LVAD) can effectively reduce cutting forces and temperatures while improving surface quality [4,5,6]. The tool–workpiece interaction mechanism differs significantly between LVAD and CD, leading to distinct axial forces and machining performance. Establishing an accurate theoretical model for drilling forces is essential for force prediction and parameter optimization.

Allotta et al. [7] established an axial force model based on metal cutting theory but neglected the chisel edge. Lee et al. [8] developed a model considering both main cutting edges and the chisel edge, although it contained many undetermined coefficients. Lin et al. [9] and Sugita et al. [10] introduced bone density and mechanistic modeling, respectively. Sui and Sugita [11] identified the relationships between rotational speed, feed rate, and drilling force through experiments. Cortical bone produces discontinuous chips, and chip clogging increases thrust force with drilling depth [12,13]. More importantly, bone is anisotropic, so the drilling direction strongly affects force magnitude. However, existing bone drilling force models are mostly derived from metal cutting theory and do not quantitatively incorporate bone anisotropy into the constitutive relation.

In this study, a mechanistic drilling force model is established for LVAD of bone, which simultaneously considers contributions from the main cutting lips, the chisel edge, and asymmetric chip thickness under non-vertical drilling. Bone anisotropy is quantitatively incorporated into the model using a direction-dependent shear strength coefficient, rather than only being discussed qualitatively. Force characteristics in yz-plane and xz-plane drilling are revealed, clarifying the combined effects of bone anisotropy and drilling orientation on axial and lateral forces.

A series of drilling experiments are conducted on porcine femurs to calibrate and validate the proposed model. The effects of feed rate, spindle speed, drill diameter, vibration parameters, and drilling angle on drilling forces are investigated. This study provides theoretical and experimental support for the optimization of drilling parameters in clinical orthopedic surgery.

## 2. Mechanistic Model of Bone Drilling

### 2.1. Kinematics Analysis

In conventional drilling (CD) and low-frequency vibration-assisted drilling (LVAD), the motion trajectory equation of the outer point on the main cutting edge was written as

(1) $$\left\{\begin{array}{l} x=r\cos\theta \\ y=r\sin\theta \\ z=-f_{r}\theta /2\pi \end{array}\right.$$

(2) $$\left\{\begin{array}{l} x=r\cos\theta \\ y=r\sin\theta \\ z=-f_{r}\theta /2\pi +A\sin(\mathrm{f\theta}/n) \end{array}\right.$$

where *r* (mm) is the radius of the drill bit, *f_r_* (mm/r) is the feed per revolution (*f_r_* = 60*v_f_*/*n, v_f_* is the feed rate (mm/s)), *θ* (rad) is the angular displacement of the drill bit (*θ* = 2*πnt*), *n* (rpm) is the rotation speed, *f* (Hz) is the vibration frequency, and *A* (μm) is the vibration amplitude.

**Figure 1:**
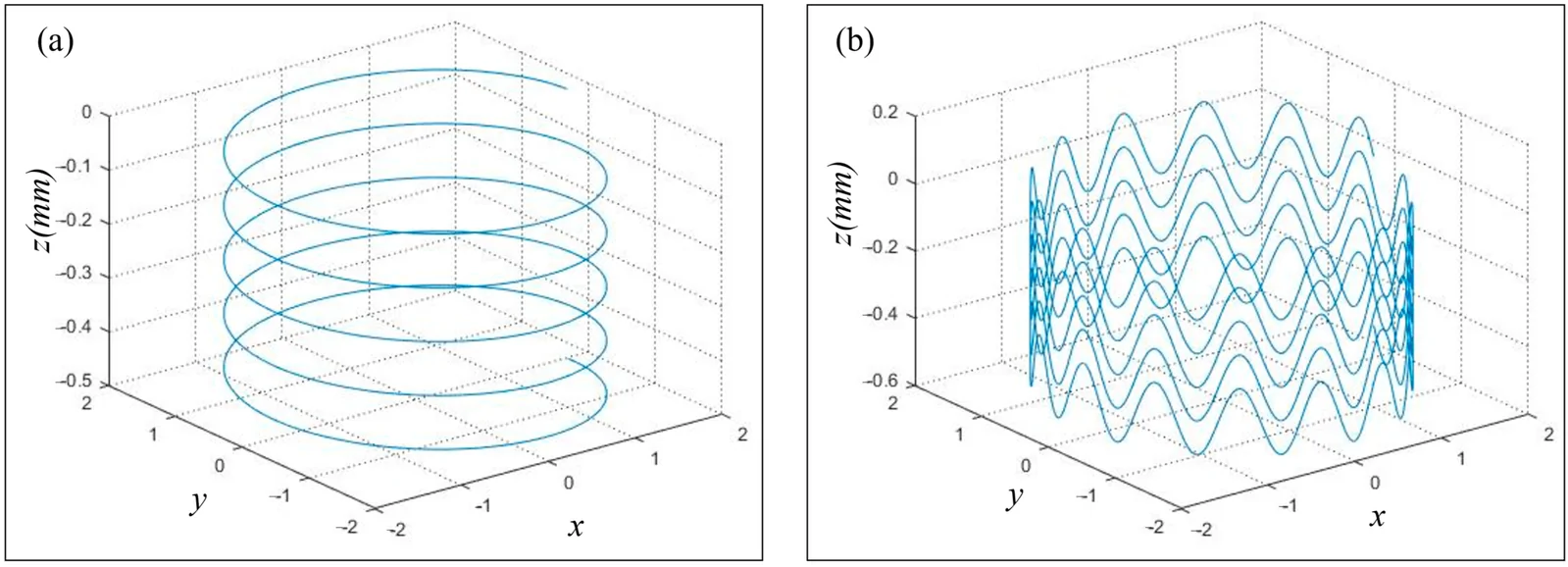
The trajectory of cutting edge in (**a**) conventional drilling (**b**) low-frequency vibration-assisted drilling.

Figure 1 shows the trajectory comparison of the cutting edge outer point between CD and LVAD. The three-dimensional trajectory of the cutting edge is a spiral curve with uniform pitch and diameter. In CD, the trajectory is a smooth continuous curve determined only by feed rate. In LVAD, the trajectory is a periodic sinusoidal curve affected by spindle speed, feed rate, vibration frequency, and amplitude.

Due to the periodic axial displacement of the cutting edge in LVAD, the cutting speed changes periodically. The instantaneous axial feed rate can be expressed as

*v_z_* = −*f_r_n* + 2*πfA*cos(2*πft*) (3)

The circumferential speed at a distance r from the spindle center is given by

*v_x_* = 2*πrn*/60 (4)

**Figure 2:**
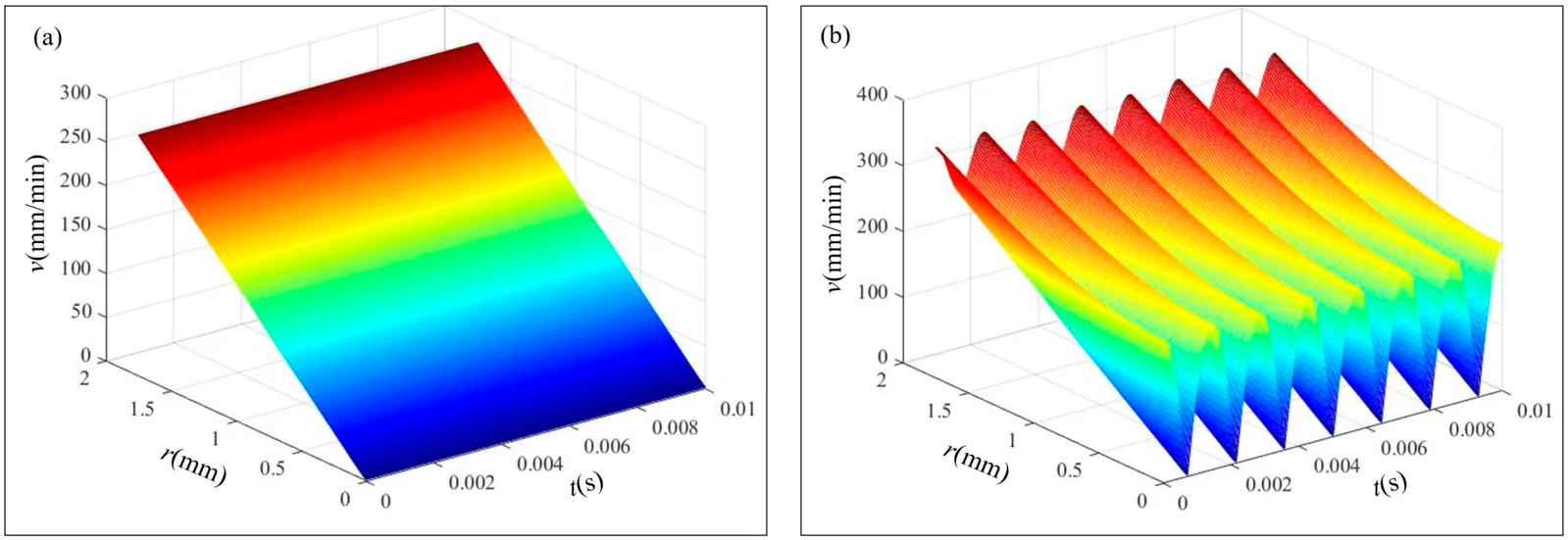
The influence of drilling method on cutting velocity (**a**) CD (**b**) LVAD.

During drilling, the actual cutting speed is the vector combination of the circumferential speed and the axial feed speed. As shown in Figure 2, the cutting speed in CD increases linearly with radius and remains stable over time. In LVAD, both the magnitude and direction of cutting speed vary periodically, and the speed at each point depends on its radial distance from the spindle center.

### 2.2. Drilling Force Model

**Figure 3:**
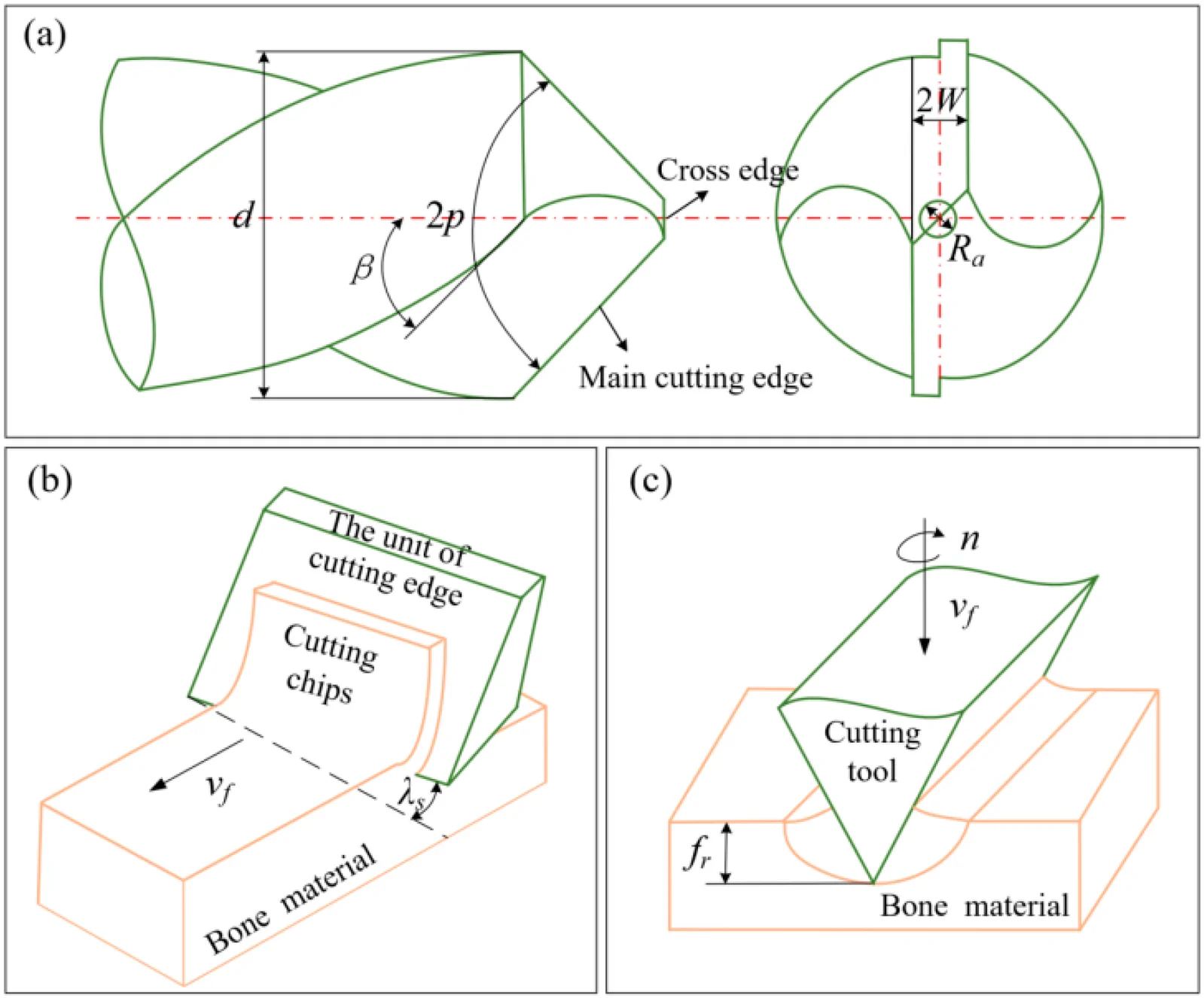
The drill bit (**a**) geometric structure (**b**) oblique cutting model (**c**) right angle cutting model.

Based on the geometric characteristics of the medical twist drill, the total drilling force is generated by two parts: the main cutting lips and the chisel edge. The drill bit is divided into a series of infinitesimal cutting elements, as shown in Figure 3. Each element on the main cutting lip is equivalent to an oblique cutting model, while the chisel edge element is treated as an orthogonal cutting/indentation model.

#### 2.2.1. Force Model Formulation for the Main Cutting Lips

**Figure 4:**
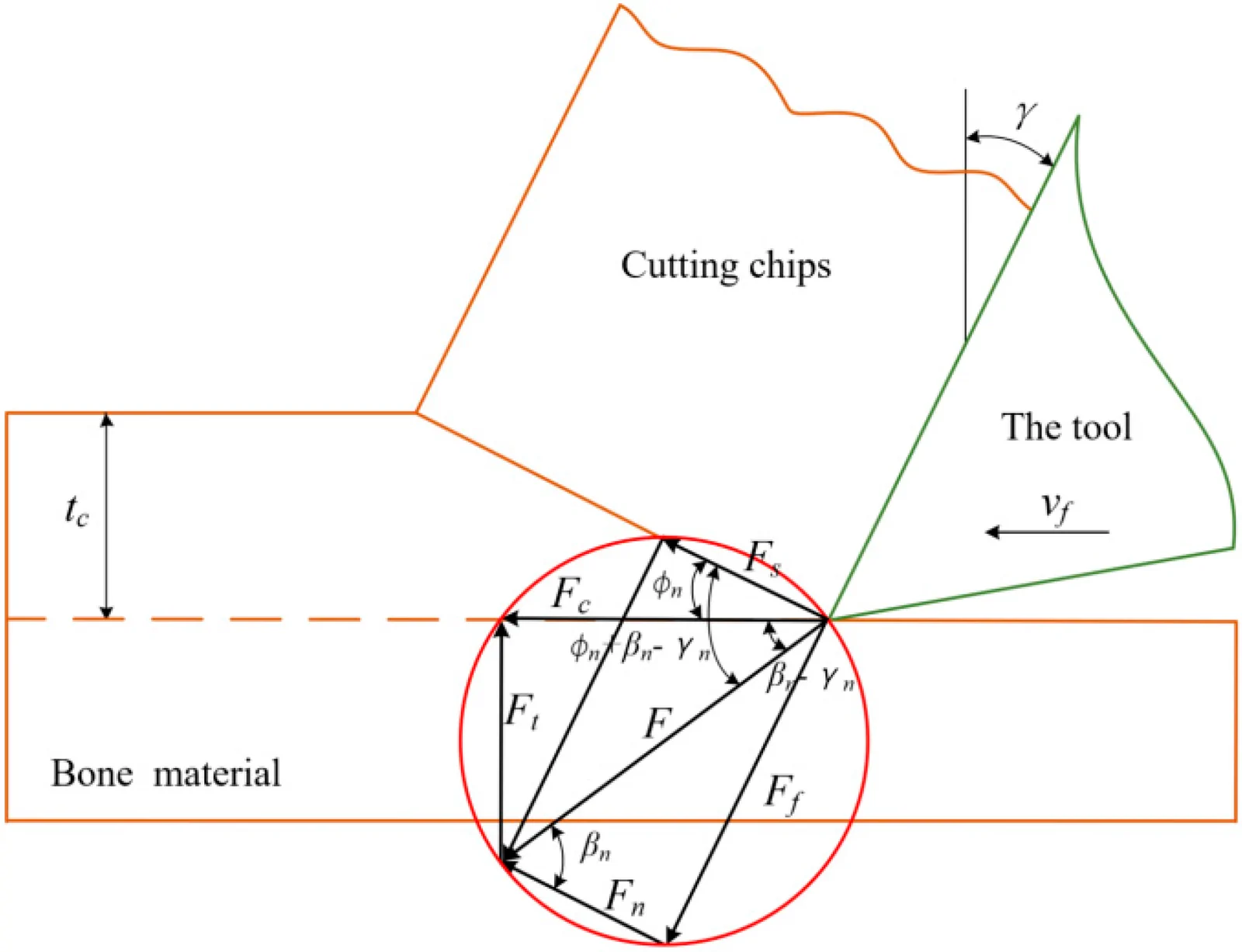
The relationship between cutting force and cutting angle.

In material removal, the cutting energy is mainly consumed in the shear deformation zone. The main cutting edge is decomposed into small units, each regarded as an oblique cutting process. The force components and geometric angles are illustrated in Figure 4.

In Figure 4, *F_s_* is the shear force, *F_n_* is the normal force, and *F_f_* is the friction force. *F_c_* is the cutting component in the direction of cutting motion. *F_t_* is the cutting component perpendicular to the cutting direction. *F* is the resultant force, and *t_c_* is the thickness of the cut chip; *γ* is the front corner of the tool, *ϕ_n_* is the shear angle, *γ_n_* is the normal forward angle, and *β_n_* is the friction angle.

In the equivalent plane, the shear force can be obtained from the material stress and shear area in the shear zone, so the unit shear force *dF_s_* along the shear plane is:

*dF_s_* = *τ_s_dA_s_* (5)

where *dA_s_* is the shear area of each cutting edge unit, and *τ_s_* is the shear yield stress of bone.

According to Davidson and James [14], the shear yield stress considering bone anisotropy is defined as:

*τ_s_*(*θ*) = *C*_1_·*K*(*θ*)·*v_c_* + *C*_2_ (6)

where *C*_1_ and *C*_2_ are constant coefficients identified by drilling experiments. *θ* denotes the drilling orientation with respect to the bone microstructure. *K*(*θ*) is the anisotropic coefficient used to characterize direction-dependent shear strength, which is calibrated by minimizing the squared error between predicted axial thrust force and experimental measurements. *K*(*θ*) = 1.0 is taken as the reference value for yz-plane drilling. For xz-plane drilling, the optimal range *K*(*θ*) = 0.7–0.9 is obtained via fitting against xz-plane oblique drilling experimental data. Notably, these fitting datasets are not reused for model validation. *v_c_* is the cutting speed (*v_c_* = 2πrn/60).

*γ_nd_* is the dynamic normal rake angle. It describes the instantaneous effective rake angle modified by the relative vibration motion between drill bit and bone workpiece in vibration-assisted drilling [15]. *γ_n_* is the static normal rake angle measured in the plane normal to the cutting edge, which can be expressed by

(7) $$\gamma_{n}=\arctan\left(\frac{\sqrt{r^{2}-t^{2}}\tan\delta }{r\sin p-t\cos p\tan\delta }\right)-\arctan\left(\frac{t\cos p}{\sqrt{r^{2}-t^{2}}}\right)$$

where *t* is half the thickness of the web plate. *p* is the half tip angle of the drill tip. *δ* is the angle between the main cutting edge and the drill bit axis ($\delta =\arctan(r_{d}\tan p/\sqrt{{r_{d}}^{2}-t^{2}})$). *δ_d_* is the angle between the cutting speed and the machining surface, which can be expressed by

(8) $$\delta_{d}=\arctan(\frac{v_{v}}{v_{x}})=\arctan(\frac{-f_{r}n+2\mathrm{\pi fA}\cos(2\mathrm{\pi ft})}{2\mathrm{\pi rn}/60})$$

According to Ernst and Merchant’s minimum energy theory [16], the shear angle *ϕ_nd_* is defined as:

(9) $$\phi_{\mathrm{nd}}=\frac{\pi }{4}+\frac{\gamma_{\mathrm{nd}}-\beta_{\mathrm{nd}}}{2}$$

where *β_nd_* is the friction coefficient between the chip and the tool (*μ*) (*β_nd_* = tan *μ*).

The chip thickness *t_c_* is:

(10) $$t_{c}=\frac{f_{r}}{2}\sin p$$

The shear area *dA_s_* of each cutting edge unit is:

(11) $$dA_{s}=\frac{t_{c}(d-2w)\cos\lambda_{s}}{\sin p\sin\phi_{\mathrm{nd}}}$$

According to the geometric relationship between cutting force and angle shown in Figure 4, the relationships between the frictional force *dF_f_*, the normal force *dF_n_*, and the shear force *dF_s_* are as follows:

(12) $$\begin{array}{l} dF_{f}=dF_{s}\frac{\sin\beta_{\mathrm{nd}}}{\cos(\phi_{\mathrm{nd}}+\beta_{\mathrm{nd}}-\gamma_{\mathrm{nd}})}\\ dF_{n}=dF_{s}\frac{\cos\beta_{\mathrm{nd}}}{\cos(\phi_{\mathrm{nd}}+\beta_{\mathrm{nd}}-\gamma_{\mathrm{nd}})} \end{array}$$

**Figure 5:**
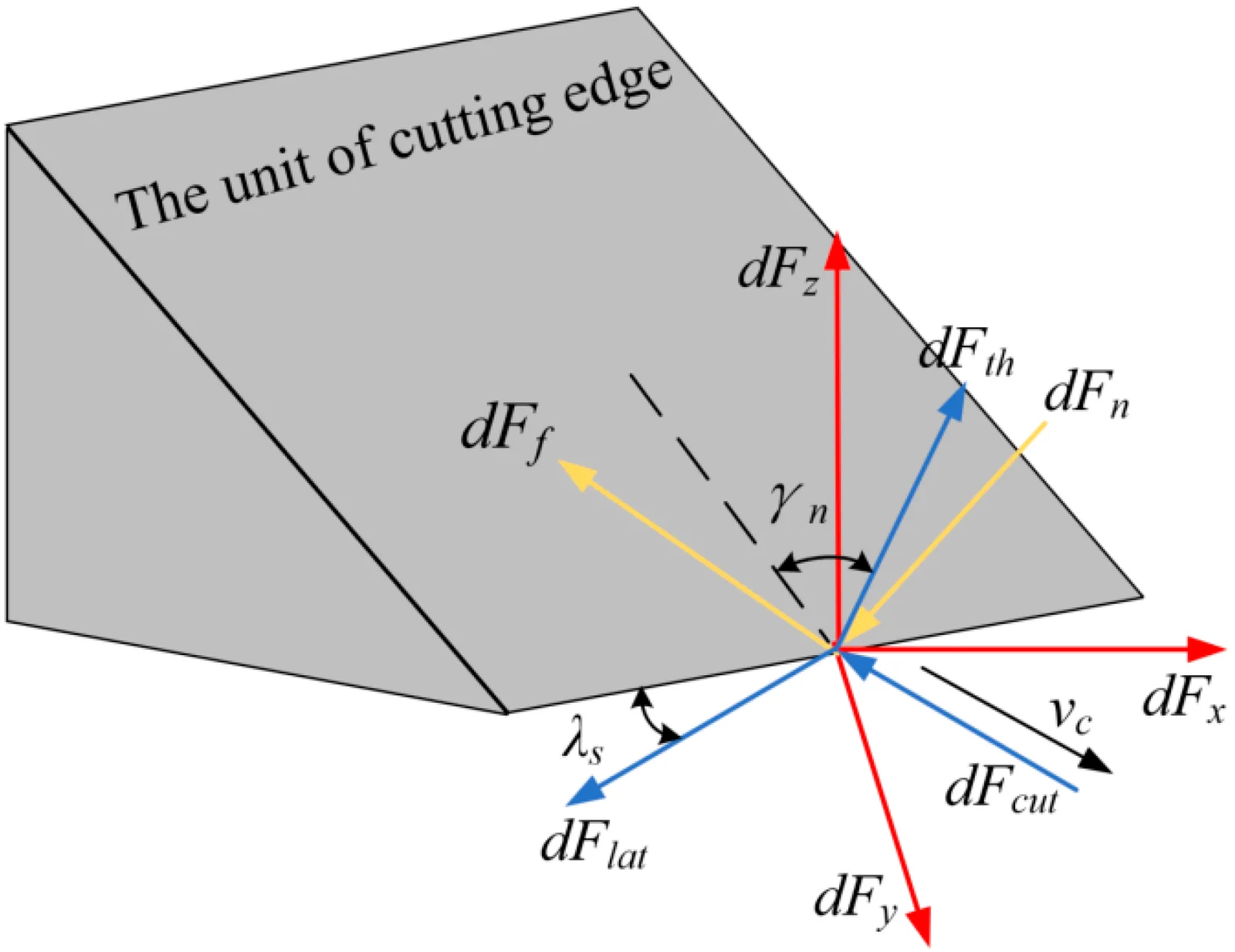
The cutting force component on the unit cutting edge.

As shown in Figure 5, the bevel cutting unit has three force components, which are the feed force *dF_th_*, the cutting force *dF_cut_* and the lateral force *dF_lat_*. They are related to the normal force and the friction in right angle cutting. The friction force and normal force in the right angle cutting model are converted into the feed force, cutting force and lateral force in the oblique cutting model through the transformation matrix as follows [8]:

(13) $$\left[\begin{array}{l} dF_{\mathrm{th}}\\ dF_{\mathrm{cut}}\\ dF_{\mathrm{lat}} \end{array}\right]=\left[\begin{array}{c} \cos\eta_{c}\cos\gamma_{\mathrm{nd}}\;\;\;\;\;\;\;\;-\sin\gamma_{\mathrm{nd}}\\ \sin\eta_{c}\sin\lambda_{s}+\cos\eta_{c}\cos\lambda_{s}\sin\gamma_{\mathrm{nd}}\;\cos\lambda_{s}\cos\gamma_{\mathrm{nd}}\\ \cos\eta_{c}\sin\lambda_{s}\sin\gamma_{\mathrm{nd}}-\sin\eta_{c}\cos\lambda_{s}\;\sin\lambda_{s}\cos\gamma_{\mathrm{nd}} \end{array}\right]\left[\begin{array}{l} dF_{f}\\ dF_{n} \end{array}\right]$$

Then

(14) $$\left\{\begin{array}{l} dF_{\mathrm{th}}=dF_{f}\cos\eta_{c}\cos\gamma_{\mathrm{nd}}-dF_{n}\sin\gamma_{\mathrm{nd}}\\ dF_{\mathrm{cut}}=dF_{f}(\sin\eta_{c}\sin\lambda_{s}+\cos\eta_{c}\cos\lambda_{s}\sin\gamma_{\mathrm{nd}})+dF_{n}\cos\lambda_{s}\cos\gamma_{\mathrm{nd}}\\ dF_{\mathrm{lat}}=dF_{f}(\cos\eta_{c}\sin\lambda_{s}\sin\gamma_{\mathrm{nd}}-\sin\eta_{c}\cos\lambda_{s})+dF_{n}\sin\lambda_{s}\cos\gamma_{\mathrm{nd}} \end{array}\right.$$

where *λs* is the blade inclination angle and *η_c_* is the chip flow angle. According to the chip direction rule derived by Stabler et al. [17] (*η_c_* = *λ_s_*), the blade inclination angle is

(15) $$\lambda_{s}=\arcsin(\frac{t}{r}\sin p)$$

The cutting force components *F_x_*, *F_y_*, and *F_z_* of the cutting edge unit in three directions during oblique cutting can be calculated as follows

(16) $$\left\{\begin{array}{l} dF_{x}=dF_{\mathrm{thu}}\frac{-\cos\beta \cos p}{\cos\lambda_{s}}+dF_{\mathrm{cut}}\sin\beta -dF_{\mathrm{lat}}\frac{\cos^{2}\beta \sin p}{\cos\lambda_{s}}\\ dF_{y}=dF_{\mathrm{thu}}\frac{-\sin\beta \cos p}{\cos\lambda_{s}}-dF_{\mathrm{cut}}\cos\beta -dF_{\mathrm{lat}}\frac{\sin\beta \cos\beta \sin p}{\cos\lambda_{s}}\\ dF_{z}=dF_{\mathrm{thu}}\frac{\cos\beta \sin p}{\cos\lambda_{s}}-dF_{\mathrm{lat}}\frac{\cos p}{\cos\lambda_{s}} \end{array}\right.$$

where *F_thu_* is the threshold thrust force required to trigger shear failure of cortical bone under the drill cutting edge.

Therefore, by integrating the axial force (*F_z_*) of each cutting edge unit, the axial thrust force on the two main cutting edges can be obtained as

(17) $$F_{1}=2\int_{r_{0}}^{r}dF_{z}$$

#### 2.2.2. Force Model Formulation for the Chisel Edge

There are a secondary cutting edge and an indentation area on the chisel edge. Because the cutting speed of the secondary cutting edge is lower than that of the main cutting edge, the drilling force generated is also lower. Therefore, the drilling force in the indentation area of the chisel edge is mainly analyzed. The indentation area is assumed to be a rigid wedge, which is pressed into the bone tissue material, and the material is extruded on both sides of the wedge, as shown in Figure 3c. According to the mathematical model of the geometric shape of the indentation area of Bono et al. [18], the force generated in the indentation area is

(18) $$F_{2}=\frac{4k_{s}(1+\varsigma )f_{r}R_{a}\sin\gamma_{\mathrm{nd}}}{\cos\gamma_{\mathrm{nd}}-\sin(\gamma_{\mathrm{nd}}-\varsigma )}$$

where *k_s_* is the shear yield strength of the material (*k_s_* = 112 MPa) [19]. *f_r_* is the feed rate, *R_a_* is the radius of the indentation zone [20], and the calculation formula is:

(19) $$R_{a}=\frac{f_{r}}{2\tan(\frac{\pi }{2}-p)}$$

*ς* is the characteristic angle obtained from the slip-line field solution. This slip-line solution *ς* acts as the determining value for calculating the slip-line angle *α_n_*, which can be calculated by

(20) $$2\alpha_{n}=\varsigma +\arccos[\tan(\frac{\pi }{4}-\frac{\varsigma }{2})]$$

#### 2.2.3. Non-Vertical Drilling

In non-vertical drilling, the chip thickness will no longer be the same as that of vertical cutting due to the deflection of the axis at the contact between the drill bit and the workpiece [21]. When the deviation angle of the drill bit to the right is *α*, the chip thickness along the right cutting edge increases, while the chip thickness along the left cutting edge decreases. Therefore, the dynamic chip thicknesses formed by the two main cutting edges are

*t_cd_*_1_ = *t_c_* + Δ*t_c_* (21)

*t_cd_*_2_ = *t_c_* − Δ*t_c_* (22)

(23) $$\Delta t_{c}=(\frac{w}{\tan p}+z)[\tan(p+\alpha )-\tan p]\cos(p+\alpha )$$

**Figure 6:**
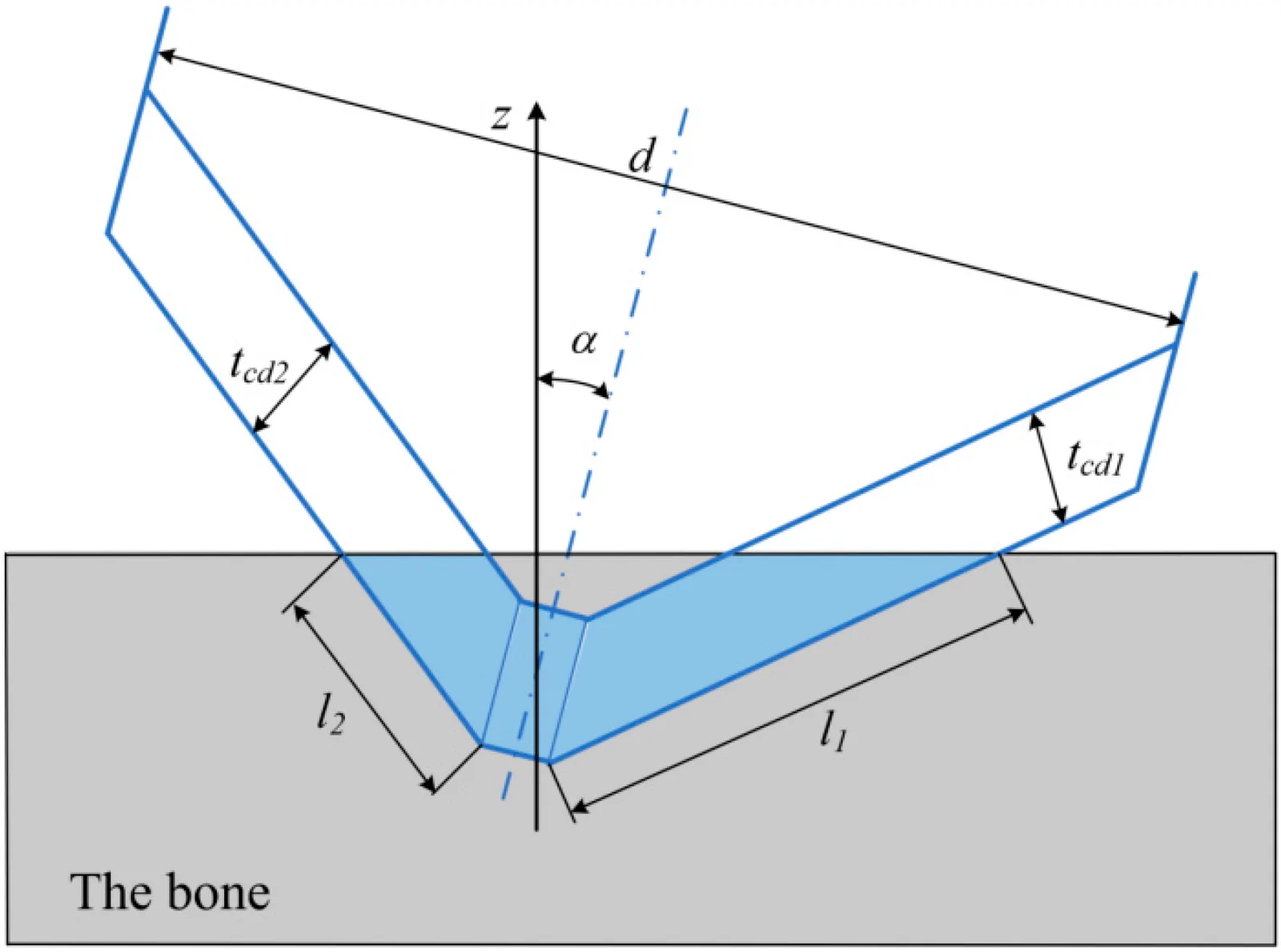
The changes in dynamic cutting area.

The dynamic chip area depends not only on the dynamic chip thickness, but also on the cutting edge length of the cutting material. For vertical drilling, the cutting lengths of the two main cutting edges are equal. However, if the surface of the workpiece is not perpendicular to the axis of the drill, the length of the main cutting edge involved in the cutting is not equal, as shown in Figure 6.

In the process of non-vertical drilling, the interaction force between the two main cutting edges on the drill and the workpiece is not equal, that is, the workpiece produces unequal cutting force on each cutting edge [22]. The dynamic cutting area generated by the cutting of the two main cutting edges is

(24) $$dA_{s1}=t_{\mathrm{cd}1}\frac{l_{1}\cos\lambda_{s}}{\sin\phi_{\mathrm{nd}}}$$

(25) $$dA_{s2}=t_{\mathrm{cd}2}\frac{l_{2}\cos\lambda_{s}}{\sin\phi_{\mathrm{nd}}}$$

where *l*_1_, *l*_2_ are the actual contact lengths between the two main cutting edges and the cortical bone, respectively. According to the simplified cutting model of the main cutting edge of the drill bit (Figure 6), the contact length is

(26) $$l_{1}=\frac{\frac{f_{r}\theta }{2\pi }\tan p+w}{\sin p(1-\sin\theta \tan\alpha \tan p)}-w\cot p$$

(27) $$l_{2}=\frac{\frac{f_{r}(\theta +\mathrm{\pi )}}{2\pi }\tan p+w}{\sin p(1+\sin\theta \tan\alpha \tan p)}-w\cot p$$

where *w* is the half-width of the drill margin. Therefore, the total cutting area of the two main cutting edges in non-vertical drilling is

*dA_s_* = *dA_s_*_1_ + *dA_s_*_2_ (28)

#### 2.2.4. Total Drilling Force

By adding the drilling forces generated on the main cutting edges and the chisel edge in the drilling process, the total drilling force can be obtained

*F* = *F*_1_ + *F*_2_ (29)

Combined with the above formulas, the theoretical model of the drilling force generated in low-frequency vibration-assisted bone drilling is established, and the prediction results can be obtained by simulation in Matlab (Version 2016b). The main factors affecting the drilling force are spindle speed *n*, feed speed *v_f_*, drill diameter *d*, drilling angle *α*, vibration frequency *f* and amplitude *A*. Therefore, under the condition of low-frequency vibration assistance, the bone drilling experiment is carried out, and the experimental results of drilling force are compared with the predicted results to verify the correctness of the model.

## 3. Experimental Materials and Methods

### 3.1. Materials and Devices

**Figure 7:**
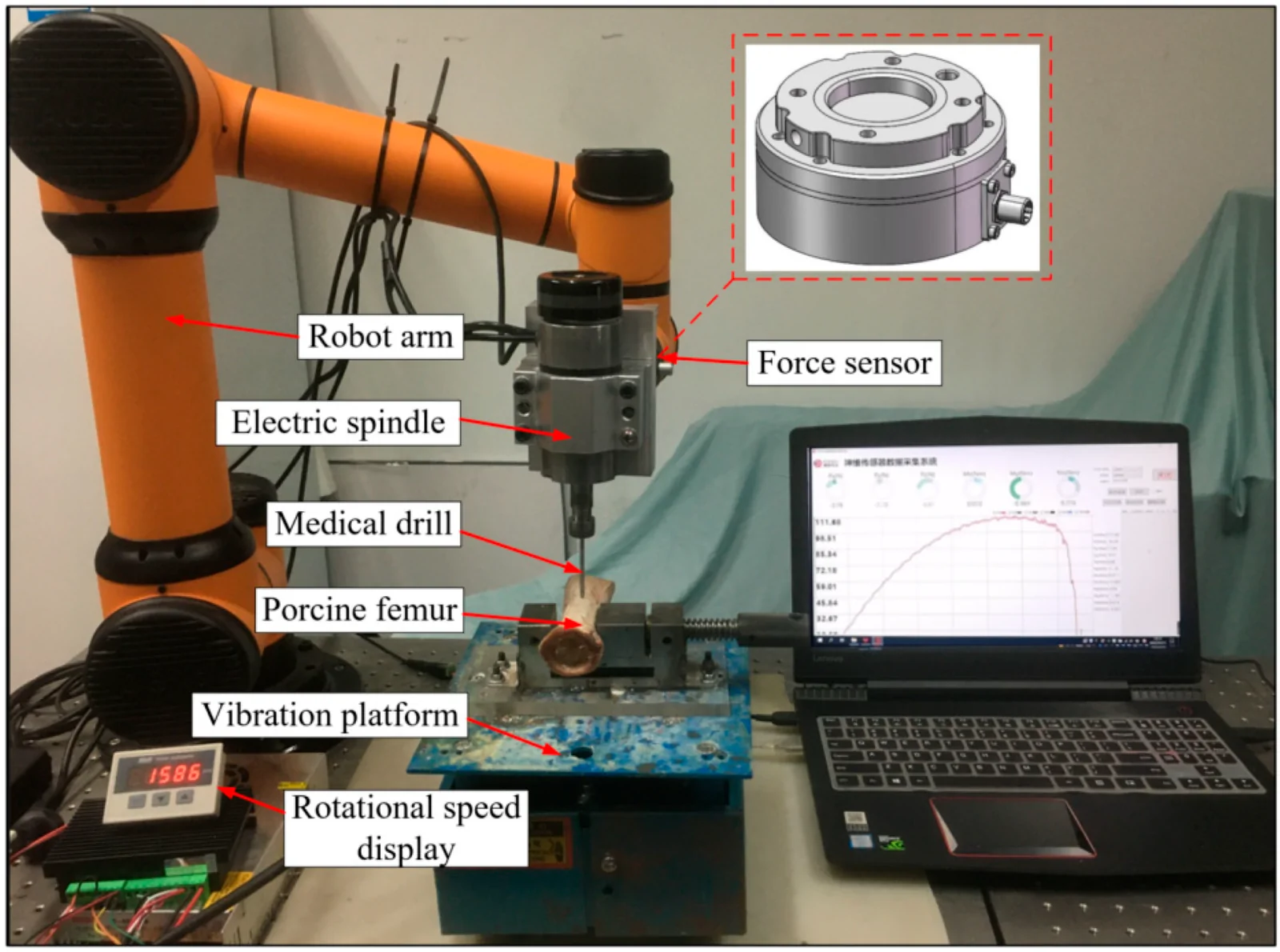
The platform of drilling experiment.

The drilling experimental platform is shown in Figure 7. Fresh porcine femurs were used as experimental material since their mechanical properties are comparable to human cortical bone. The specimens were harvested from healthy adult pigs sourced from a local commercial slaughterhouse, and no animals were sacrificed specifically for this study. A 6-degree-of-freedom robotic arm (AUBO-i5, Aubo Intelligent Technology Co., Ltd., Beijing, China) was employed to provide accurate positioning and controllable feed rate during drilling, with a positioning accuracy of ±0.02 mm. An electric spindle (ER11-48V-400W, Hengqu Electrical Machinery Co., Ltd., Shenzhen, China) was adopted to drive the medical twist drill, with a diameter of 3.5 mm and a length of 70 mm.

Drilling forces were measured using a strain-type six-axis force/torque sensor (KWR75B, Changzhou Kunwei Sensing Technology Co., Ltd., Changzhou, China), which simultaneously recorded the axial force *F_z_* and lateral forces *F_x_*, *F_y_*. The data sampling frequency was set to 5 kHz to ensure sufficient resolution for capturing dynamic vibration signals. A second-order Butterworth low-pass filter was used to suppress high-frequency electrical noise, while preserving dynamic force fluctuations originating from low-frequency vibration-assisted drilling. The filter cut-off frequency was selected empirically to remove noise without distorting vibration-related force responses. Transient signal portions for drill entry and exit were removed. Only steady-state signal segments within cortical bone were selected. The reported drilling force corresponds to the maximum value of axial thrust force within the steady-state interval.

A vertical low-frequency vibration platform (LD-20W, Guangzhou Fanzheng Electromagnetic Technology Co., Ltd., Guangzhou, China) was used to provide vibration excitation. The vibration frequency can be adjusted from 150 Hz to 2000 Hz, and the input voltage ranges from 0 to 220 V.

An acceleration sensor (Kistler 8763B050BB, Kistler Instrumente AG, Winterthur, Switzerland) was used to measure the vibration amplitude. Signals were collected by a data acquisition card (NIDAQ-6211, National Instruments Corporation, Austin, TX, USA) combined with LabVIEW software (Version 2022), and post-processed in Matlab.

**Figure 8:**
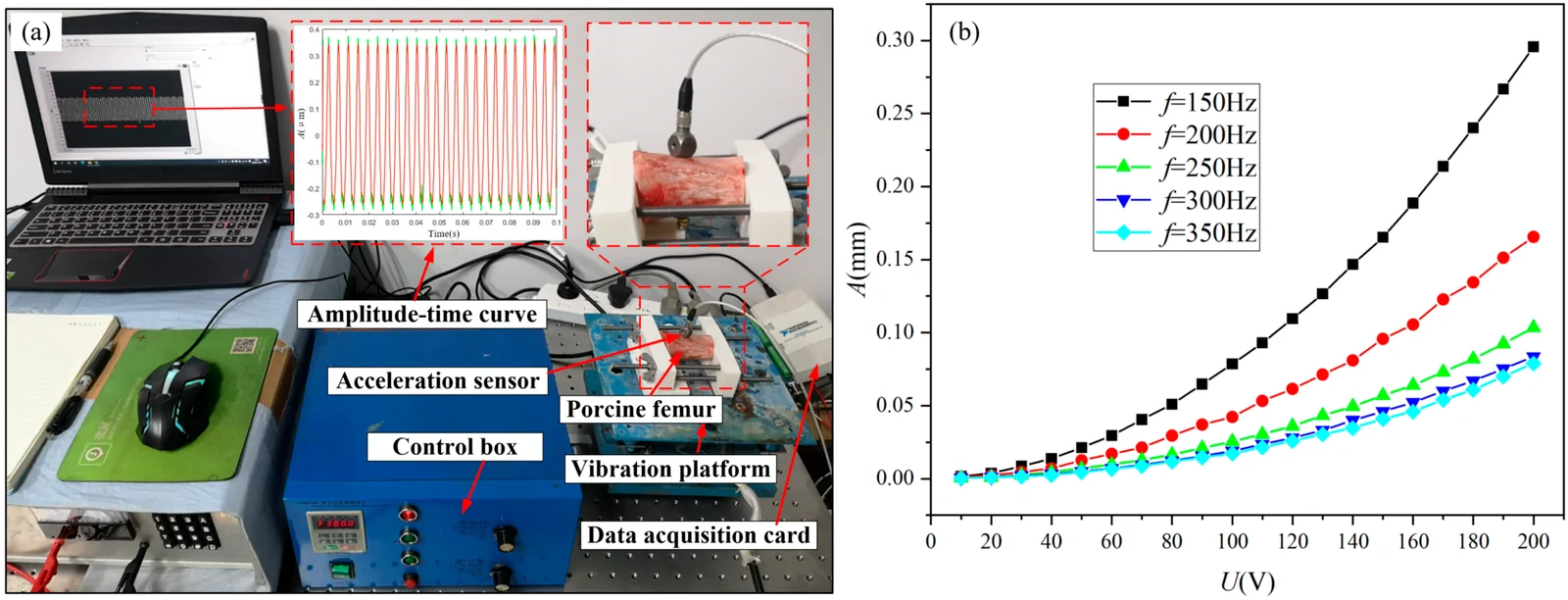
The amplitude of vibration platform: (**a**) the measurement device; (**b**) the variation in amplitude.

According to the measured data collected, the relationship between the input voltage, the vibration frequency and the amplitude is obtained as shown in Figure 8. It can be seen that the vibration amplitude increased with the increase in input voltage and decreased with the increase in vibration frequency. Therefore, the input voltage U and vibration frequency are used as vibration parameters in this study.

**Figure 9:**
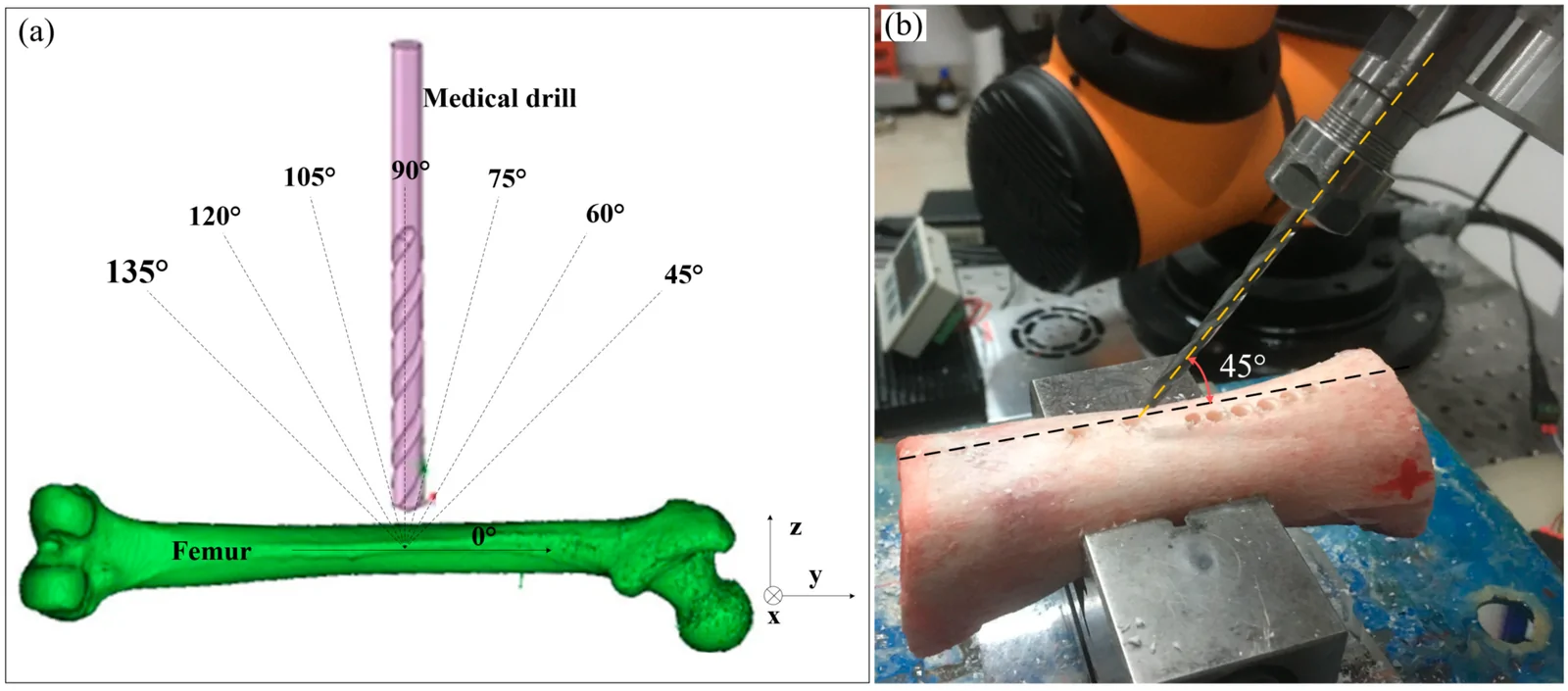
The drilling angles during the experiment: (**a**) *α* = 45°~135°; (**b**) *α* = 45°.

Clinical orthopedic surgery often requires non-vertical bone drilling, which introduces lateral forces and may cause drill slippage and eccentricity. The drilling angle *α* is defined as the angle between the drill axis and the bone surface normal. The drilling angle was varied from 45° to 135° by adjusting the posture of the robotic arm (Figure 9). Due to the anisotropic nature of cortical bone, the influences of drilling orientation and angle on axial and lateral forces were investigated systematically.

The yz-plane and xz-plane were defined based on the macroscopic geometry of porcine femoral diaphysis. The yz-plane contains the long axis of the femoral shaft, and the xz-plane is perpendicular to this long axis. Specimens were manually positioned according to bone outer morphology to achieve consistent orientation across samples. It should be noted that osteonal microstructures were not characterized via micro-CT or histology in this study.

### 3.2. Experimental Design

Drilling experiments were carried out using the single-factor experimental method to investigate the effects of machining parameters on drilling forces. The parameter levels are listed in Table 1. All tests were performed under dry cutting conditions. Each test was repeated three times, and the average value was taken as the final result to reduce random error. For comparison, both conventional drilling (CD) and low-frequency vibration-assisted drilling (LVAD) were conducted. In CD, the input voltage and vibration frequency were set to 0.

To eliminate the influence of chip clogging and drilling depth on thrust force, all tests were carried out at a shallow drilling depth (≤5 mm). Preliminary experiments confirmed that no obvious chip accumulation or clogging occurred within this range, so the steady-state force model is valid. The experimental parameters included feed rate *v_f_*, spindle speed *n*, drill diameter *d*, input voltage *U*, vibration frequency *f*, and drilling angle *α*. The parameter ranges and levels are given in Table 1.

### 3.3. Model Calibration

To determine the coefficients *C*_1_ and *C*_2_ in Equation (5), nonlinear least-squares fitting was performed using the axial thrust force data measured from conventional drilling (CD) experiments listed in Table 2. The objective function was constructed to minimize the root-mean-square deviation between the model-predicted thrust force and the experimentally measured thrust force. Since the low-frequency vibration-assisted drilling (LVAD) model is developed based on the conventional drilling theoretical framework, the calibration of *C*_1_ and *C*_2_ was carried out using the CD dataset in Table 2. The calibrated coefficients are obtained as *C*_1_ = 76.036 and *C*_2_ = 13.51. The coefficient of determination *R*^2^ = 0.9–0.97 and the root-mean-square error RMSE < 3.2 N, indicating excellent goodness-of-fit between the fitted model and the calibration data.

It should be emphasized that the datasets used to calibrate parameters *C*_1_, *C*_2_ and anisotropic coefficient *K*(*θ*) are strictly separated from datasets used for model validation. The calibration datasets include selected conventional drilling tests for *C*_1_/*C*_2_ and partial xz-plane oblique drilling tests for *K*(*θ*). Validation is performed on completely independent experimental groups (feed rate, spindle speed, drill diameter, vibration parameters and perpendicular angle tests).

## 4. Results and Discussion

### 4.1. The Effect of Drilling Parameters

(1) The feed rate

**Figure 10:**
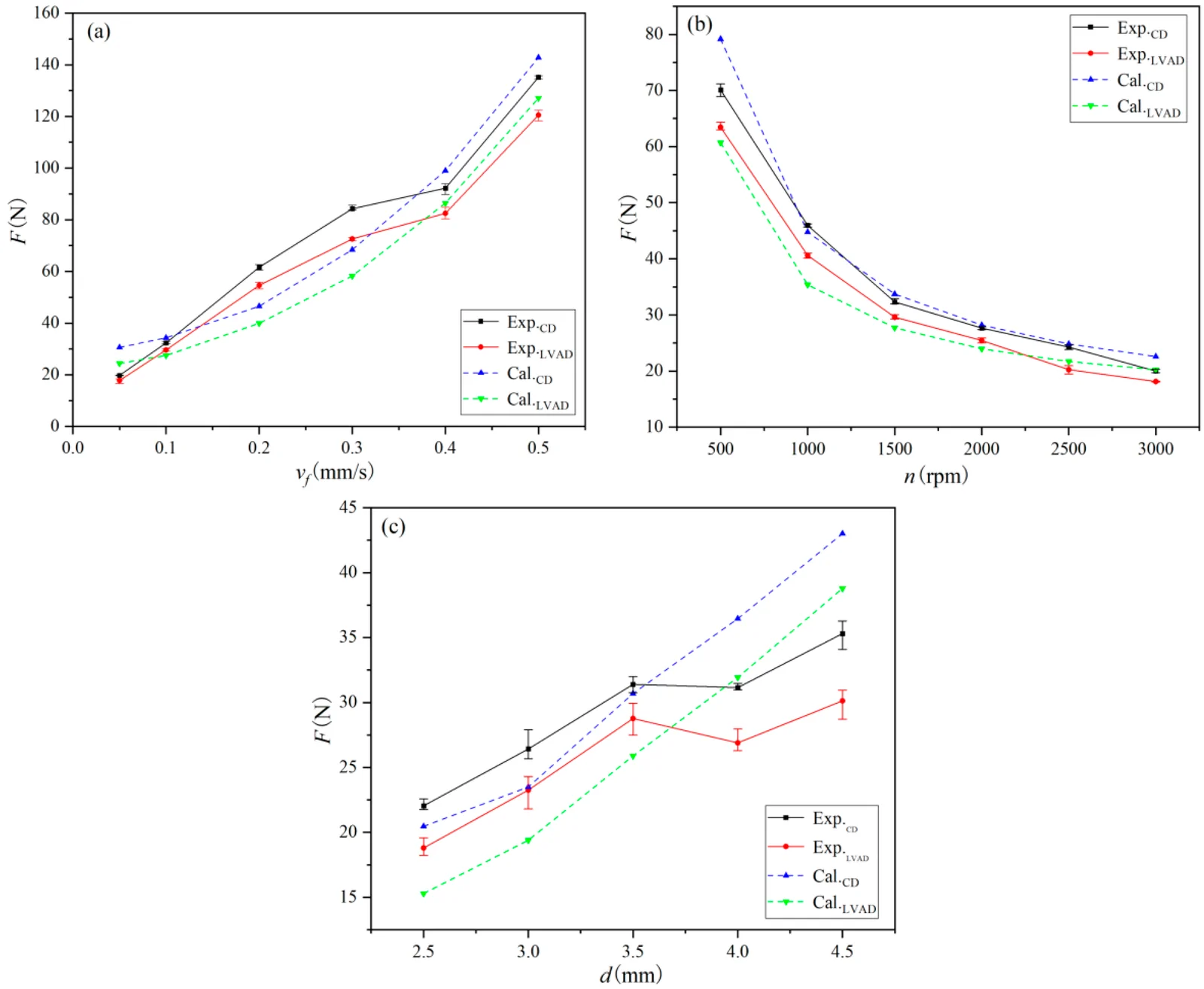
The influence of drilling parameters on the drilling force: (**a**) the feed rate; (**b**) the rotational speed; (**c**) the diameter.

The experimental results (Exp.) and predicted results (Cal.) of the drilling force generated in LVAD and CD under different feed rate conditions are shown in Figure 10a. It can be seen from the figure that with the increase in feed rate, the drilling force obtained in both processing methods increases continuously while the drilling force obtained in LVAD is lower than that in CD, and the reduction effect is more obvious when the feed rate is higher. In the process of bone drilling, with the increase in feed rate, the removal rate of bone material is increasing, which leads to the increasing resistance of the drill bit, so the drilling force is also increasing. Furthermore, due to the viscoelastic properties of bone material, the increase in feed rate will increase the area of elastic–plastic deformation, so the deformation resistance will increase, which will lead to the increase in drilling force.

Compared with CD, the drilling force generated in LVAD is lower, and the maximum and average reduction rates are 13.86% and 10.85%, respectively. In each cycle of LVAD, there is intermittent contact between the workpiece and the tool. Compared with CD, the cutting time, the average drilling thickness and the friction coefficient under the condition of vibration assistance are reduced, and the friction force is reduced, which leads to the reduction in drilling force. By comparison, it can be seen that under the same drilling parameters, with the increase in feed rate, the predicted results of drilling force and the experimental results have the same change trend, and the error is within a reasonable range, which proves the correctness and accuracy of the theoretical model.

(2) The rotational speed

The experimental and predicted results of drilling forces obtained from low-frequency vibration-assisted drilling and conventional drilling under different spindle speeds are shown in Figure 10b. As the spindle speed increases, the drilling forces in both processes decrease continuously. This decrease is attributed to the reduction in cutting depth as the spindle speed rises, leading to thinner chip thickness, lower chip shear deformation forces, and decreased friction during the drilling process, all contributing to the reduction in drilling forces. Additionally, compared to conventional drilling, lower drilling forces are observed in low-frequency vibration-assisted drilling, with maximum and average reductions of 16.52% and 11.6%, respectively. This is due to faster chip breaking in low-frequency vibration-assisted drilling, which shortens the contact time between the chips and the tool, resulting in decreased drilling forces. Therefore, the application of low-frequency vibration assistance can effectively reduce drilling forces. By comparing the results, it can be seen that under the same process parameters, the predicted and experimental drilling forces exhibit the same trend. The maximum prediction error is 12.87%, and the average prediction error is 9.6%, which are within a reasonable range, validating the correctness of the established theoretical model.

(3) The diameter

The influence curve of drill bit diameter on drilling force is shown in Figure 10c. As the drill bit diameter continues to increase, the drilling force obtained from both processing methods shows an upward trend. However, when *d* = 4 mm, the drilling force decreases slightly. When the drill bit diameter increases from 2.5 mm to 4.5 mm, the drilling force obtained in conventional drilling increases by 60.59%. As the drill bit diameter increases, the cutting width (*b_D_*) increases proportionally (*b_D_* = *d*/2sin φ), resulting in an increased cutting area of the bone material, which means a higher material removal rate per unit time, thereby increasing the generated drilling force.

Under the same processing parameters, the drilling force produced during low-frequency vibration-assisted drilling is smaller compared to conventional drilling. Therefore, the low-frequency vibration-assisted method can reduce the drilling force. Furthermore, as shown in Figure 10c, there is a certain error between the experimental and theoretical results of the drilling force. This is due to the difference between the theoretical and actual friction coefficients of drill bits of different diameters during bone drilling, which introduces some error into the experimental results. However, the experimental and theoretical values share the same upward trend, indicating that the studied model can still predict changes in the drilling force.

### 4.2. The Effect of Vibration Parameters

(1) The input voltage

**Figure 11:**
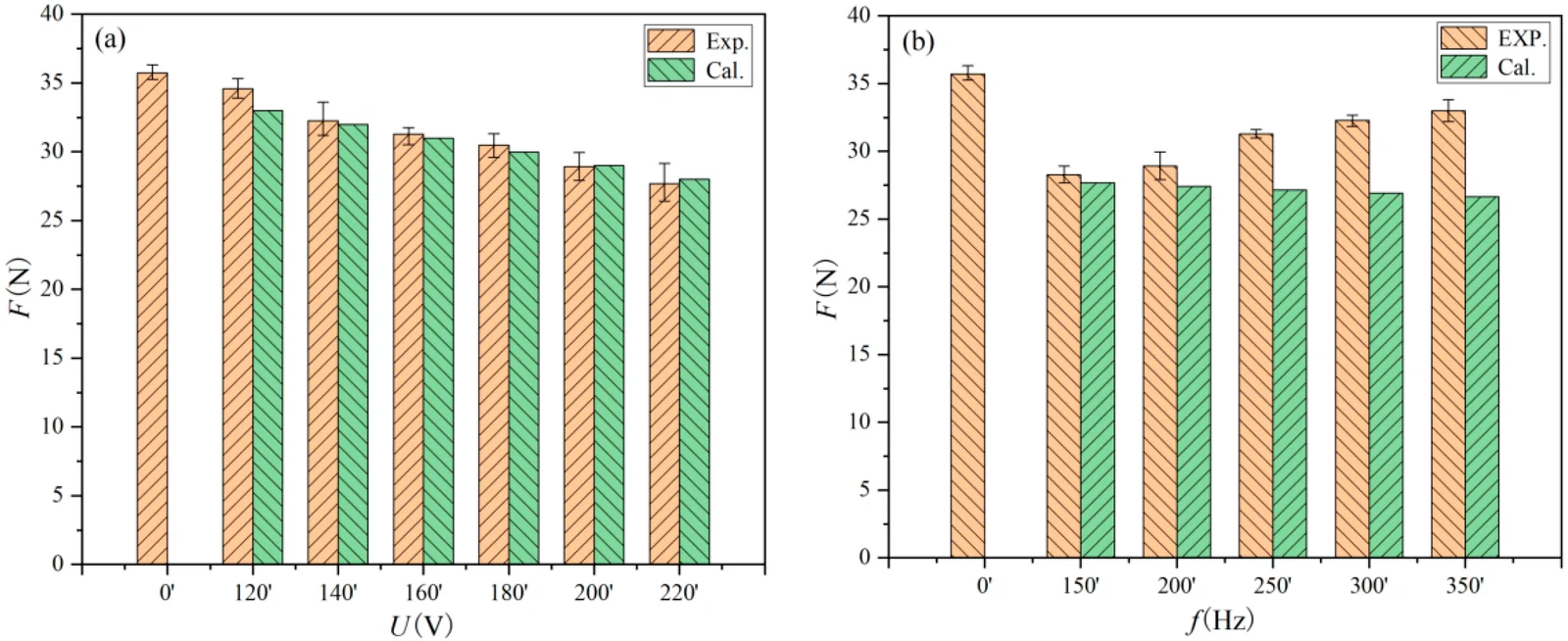
The influence of vibration parameters on the drilling force: (**a**) input voltage; (**b**) vibration frequency.

The influence curve of input voltage on drilling force is shown in Figure 11a. According to Figure 8, when the vibration frequency is fixed, a higher input voltage to the vibration platform results in a larger amplitude during the drilling process. Therefore, the level of input voltage represents the magnitude of the amplitude. When the input voltage is 0, it corresponds to conventional drilling. The figure indicates that as the input voltage (amplitude) increases, the drilling force tends to decrease. When the voltage increases from 120 V to 220 V, the decrease in drilling force is only 19.88%. With an increase in input voltage (amplitude), the contact time between the drill bit and bone tissue decreases, resulting in reduced relative cutting time and lower friction between the cutting edge and bone tissue, which leads to a decrease in drilling force. However, during the experiment, the drilling force tended to reduce the amplitude of the vibration platform, affecting the drilling performance. Therefore, the decrease in drilling force with an increase in input voltage (amplitude) was relatively small.

Furthermore, comparisons show that under the same drilling parameters, the drilling force obtained in conventional drilling is higher than that in low-frequency vibration-assisted drilling. This is due to the different contact modes; in conventional drilling, the tool and workpiece are in continuous contact, while in vibration-assisted drilling, they are in intermittent contact, leading to differences in drilling force. According to Figure 11a, as the input voltage continues to increase, the predicted and experimental results exhibit the same trend. The maximum prediction error for drilling force is 10.48%, and the average prediction error is 4.5%, which are within an acceptable range, proving the accuracy of the theoretical model.

(2) The vibration frequency

Figure 11b illustrates the variation in drilling force with vibration frequency. It can be observed that the measured drilling force increases with increasing frequency, while the predicted force shows a decreasing trend. This discrepancy arises because the vibration amplitude decreases with increasing frequency under constant input voltage (as shown in Figure 8). Therefore, the present experiment does not reflect an independent frequency effect, but rather the combined effect of frequency and amplitude.

Theoretically, if amplitude were held constant, drilling force would decrease with increasing frequency, since higher frequency induces more frequent tool–workpiece separation and reduces friction. However, in the present experimental setup, the amplitude drop dominates the trend, leading to an overall slight increase in drilling force (only 3.75% from 150 Hz to 350 Hz). Compared with conventional drilling, low-frequency vibration-assisted drilling still achieves lower drilling forces under all tested conditions.

It should be noted that the amplitude–voltage relationship shown in Figure 8 was calibrated under unloaded conditions. During actual bone drilling, cutting forces exert dynamic load on the vibration platform and damp the real vibration amplitude, and real-time amplitude was not recorded in the present experiments. Therefore, the amplitude adopted for model simulation originates from unloaded calibration data, which constitutes one source of deviation between predicted and experimental drilling force results. Furthermore, frequency and amplitude are inherently coupled within this experimental setup: under fixed input voltage, vibration amplitude decreases as frequency increases. As a result, the measured drilling force trend reflects combined effects of frequency and amplitude, rather than the pure independent effect of frequency. The theoretical prediction that higher frequency reduces drilling force holds only for constant-amplitude conditions. Owing to this coupling, current experimental results cannot serve as validation for the frequency-dependent response of the proposed model. Model validation in this study is mainly supported by tests of feed rate, spindle speed, drill diameter, vibration amplitude and drilling orientation. Both limitations suggest that future studies should implement real-time amplitude acquisition under loaded drilling conditions to improve model reliability.

### 4.3. The Effect of Drilling Angle

**Figure 12:**
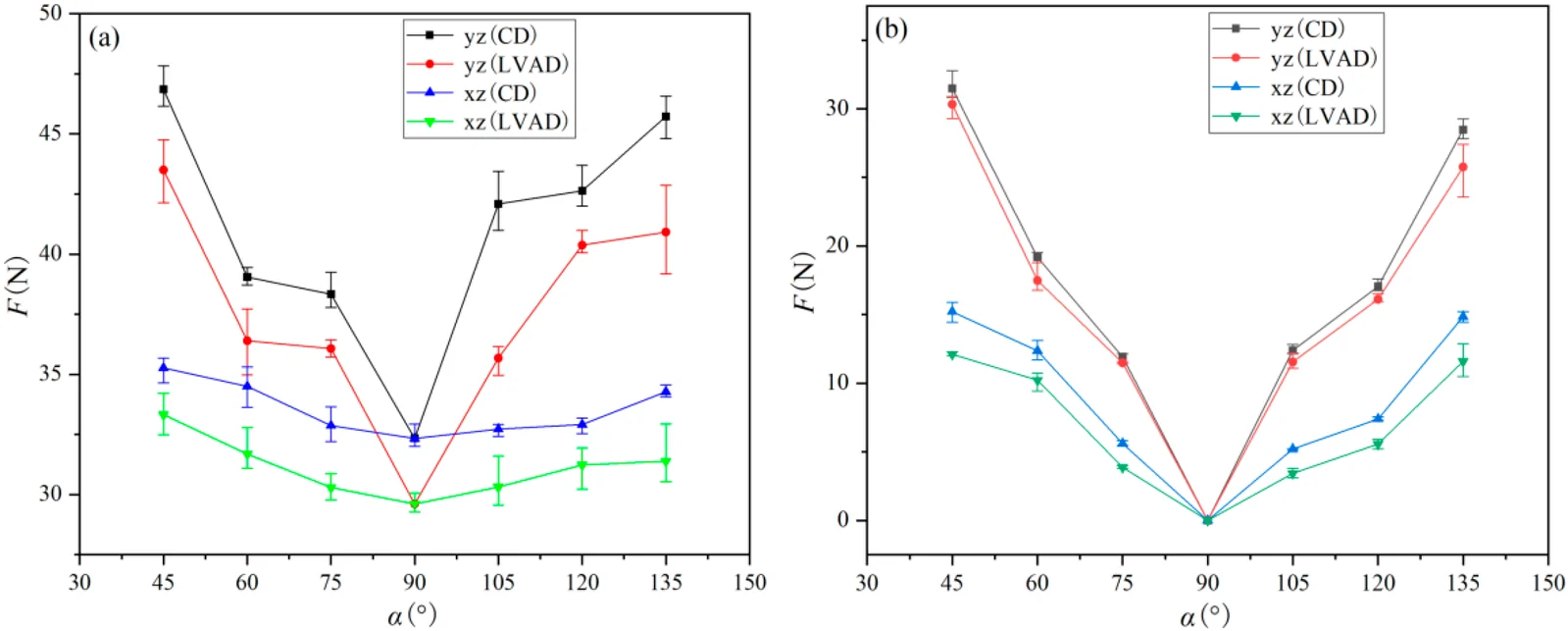
The influence of drilling angle on the drilling force: (**a**) axial force; (**b**) lateral force.

According to Liao et al. [23] in their study on orthogonal cutting of bone materials, the material deformation mechanisms differ across various cutting directions (parallel, crosswise, and perpendicular), which is related to the anisotropic characteristics of bone materials. Therefore, during bone drilling, the drilling forces generated vary at different drilling angles and directions. Based on the coordinate directions in Figure 9, drilling was conducted within the yz and xz planes, respectively, to obtain the influence of drilling angle on axial force (*F_z_*) and lateral forces (*F_y_*, *F_x_*), as shown in Figure 12. When *α* = 90°, it is perpendicular drilling; when 45° < *α* < 90° and 90° < *α* < 135°, it is non-perpendicular drilling. The figure reveals that within both the yz and xz planes, the axial and lateral forces obtained during non-perpendicular drilling are greater than those during perpendicular drilling, and the axial and lateral forces within the yz plane are both greater than those within the xz plane. Furthermore, within both the yz and xz planes, the axial and lateral forces obtained when the drilling angle is within the ranges of 45° ≤ *α* < 90° and 90° < *α* ≤ 135° are basically symmetrical on both sides relative to *α* = 90°. Additionally, within both the yz and xz planes, compared to conventional drilling, the axial and lateral forces obtained during low-frequency vibration-assisted drilling are smaller.

The variation in drilling force caused by different drilling angles is due to the fact that, during the drilling process, when the thickness of the cortical bone material is constant, changes in the drilling angle affect the drilling depth, which in turn influences the magnitude of the drilling force. According to the research by Lee et al. [8], the thickness of cortical bone affects the drilling force generated during the drilling process, as increasing the drilling thickness requires more shear energy, thereby increasing the drilling force. For example, given a cortical bone vertical thickness of 5 mm: at *α* = 90° (perpendicular drilling), the drill bit travel distance along its axis equals the vertical penetration depth (5 mm). For oblique drilling at *α* = 45°, the vertical penetration depth into bone remains 5 mm, whereas the drill bit travel distance along its tilted axis becomes 5/cos(45°) ≈ 7.07 mm.

During conventional drilling, the axial forces obtained within the yz plane are 32.33 N and 46.85 N, and the lateral forces are 0 N and 19.17 N, respectively; within the xz plane, the axial forces are 32.33 N and 35.27 N, and the lateral forces are 0 N and 15.21 N, respectively. Therefore, both the axial and lateral forces in perpendicular drilling are lower than those in non-perpendicular drilling.

When drilling within the yz and xz planes, the positional relationships between the cutting edge unit of the tool and the bone unit are parallel and perpendicular, respectively. Here, the cutting edge unit is obtained by finite decomposition of the drill bit’s cutting edge, while the bone unit is the most basic unit in the microstructure of cortical bone. The bone unit exhibits the greatest strength in the axial direction, assuming it is cylindrical and uniformly distributed along the axial direction of the bone. Within the yz plane, the feed direction of the cutting edge is parallel to the direction of the bone unit, and during the cutting process, the bone unit is removed in the form of shear fracture. Due to the high bending strength of the bone unit, more energy is consumed during the material removal process, resulting in a larger drilling force. Within the xz plane, the feed direction of the cutting edge is perpendicular to the direction of the bone unit, and during the cutting process, the bone unit is mainly stripped from the substrate by the tool, requiring less energy for material removal, and thus generating a smaller drilling force. Therefore, under the same process parameters, the axial and lateral forces obtained during drilling within the yz plane are both greater than those within the xz plane. This conclusion also verifies the anisotropic characteristics of bone materials.

## 5. Conclusions

In this study, a mechanistic drilling force model for low-frequency vibration-assisted bone drilling is developed and experimentally validated, with quantitative consideration of bone anisotropic effects. The model integrates contributions from the main cutting lips, the chisel edge, and asymmetric chip formation under non-vertical drilling conditions. A series of tests on porcine femurs are carried out to analyze the effects of feed rate, spindle speed, drill diameter, vibration parameters, drilling angle, and orientation. The main conclusions are summarized as follows:

(1) A reliable drilling force model is established for low-frequency vibration-assisted bone drilling. Drilling forces increase with feed rate and drill diameter and decrease with spindle speed and vibration amplitude. The introduction of an anisotropic coefficient enables the model to capture direction-dependent shear strength of cortical bone.
(2) Experimental results show that low-frequency vibration-assisted drilling reduces drilling forces by up to 16.52% compared with conventional drilling. The predicted and measured forces are in good agreement, with high R2 and low RMSE, which validates the accuracy of the proposed model.
(3) Drilling characteristics are affected by bone anisotropy and orientation. Perpendicular drilling (*α* = 90°) produces lower axial and lateral forces than non-perpendicular drilling. Forces in the yz-plane are remarkably higher than those in the xz-plane, which confirms the anisotropic mechanical behavior of cortical bone.
(4) Higher spindle speeds, lower feed rates, smaller drill diameters, and larger vibration amplitudes are recommended to reduce drilling forces. The proposed model can offer theoretical support for parameter-optimization-related research. Further in vitro and in vivo assessments covering drilling temperature, bone microcracks and biological responses are required before translating these results into clinical orthopedic bone drilling applications.

## Figures and Tables

**Table 1:** The process parameters and its levels during the drilling experiment.

No.	*v_f_* (mm/s)	*n* (rpm)	*d* (mm)	*U* (V)	*f* (Hz)	*α* (°)
1	0.05, 0.1, 0.2, 0.3, 0.4, 0.5	1500	3.5	0, 200	0, 200	90
2	0.1	500, 1000, 1500, 2000, 2500, 3000	3.5	0, 200	0, 200	90
3	0.1	1500	2.5, 3, 3.5, 4, 4.5	0, 200	0, 200	90
4	0.1	1500	3.5	120, 140, 160, 180, 200, 220	200	90
5	0.1	1500	3.5	200	150, 200, 250, 300, 350	90
6	0.1	1500	3.5	0, 200	0, 200	45, 60, 75, 90, 105, 120, 135

**Table 2:** Drilling parameters and experimental results in CD.

No.	*v_f_* (mm/s)	*n* (rpm)	*d* (mm)	*α* (°)	*F* (N)
1	0.1	1500	3.5	90	32.47
2	0.2	1500	3.5	90	60.53
3	0.3	1500	3.5	90	84.30
4	0.1	1000	3.5	90	45.93
5	0.1	2000	3.5	90	27.64
6	0.1	1500	3.0	90	25.69
7	0.1	1500	4.0	90	31.99
8	0.1	1500	3.5	75	38.33
9	0.1	1500	3.5	60	39.04

## Data Availability

The original contributions presented in this study are included in the article. Further inquiries can be directed to the corresponding authors.
